# An Optimized Fluorescence-Based Bidimensional Immunoproteomic Approach for Accurate Screening of Autoantibodies

**DOI:** 10.1371/journal.pone.0132142

**Published:** 2015-07-01

**Authors:** Virginie Dutoit-Lefèvre, Sylvain Dubucquoi, David Launay, Vincent Sobanski, Patricia Dussart, Philippe Chafey, Cédric Broussard, Sophie Duban-Deweer, Patrick Vermersch, Lionel Prin, Didier Lefranc

**Affiliations:** 1 Institut d’Immunologie, Centre de Biologie Pathologie et Génétique, Centre Hospitalier Régional Universitaire, Lille, France; 2 UMR 995, LIRIC, INSERM, Lille, France; 3 EA 2686, Univ Lille Nord de France, Lille, France; 4 Service de Médecine Interne et d’Immunologie clinique, Centre National de Référence Maladies rares, Centre Hospitalier Régional Universitaire, Lille, France; 5 INSERM U1016, Institut Cochin, Paris, France; 6 UMR 8104, CNRS Paris, France; 7 Plate-forme Protéomique 3P5, Univ Paris Descartes, Sorbonne Paris Cité, Paris, France; 8 LBHE, EA 2465, UArtois, Lens, France; 9 Service de Neurologie D, Centre Hospitalier Régional Universitaire, Lille, France; McGill University, CANADA

## Abstract

Serological proteome analysis (SERPA) combines classical proteomic technology with effective separation of cellular protein extracts on two-dimensional gel electrophoresis, western blotting, and identification of the antigenic spot of interest by mass spectrometry. A critical point is related to the antigenic target characterization by mass spectrometry, which depends on the accuracy of the matching of antigenic reactivities on the protein spots during the 2D immunoproteomic procedures. The superimposition, based essentially on visual criteria of antigenic and protein spots, remains the major limitation of SERPA. The introduction of fluorescent dyes in proteomic strategies, commonly known as 2D-DIGE (differential in-gel electrophoresis), has boosted the qualitative capabilities of 2D electrophoresis. Based on this 2D-DIGE strategy, we have improved the conventional SERPA by developing a new and entirely fluorescence-based bi-dimensional immunoproteomic (FBIP) analysis, performed with three fluorescent dyes. To optimize the alignment of the different antigenic maps, we introduced a landmark map composed of a combination of specific antibodies. This methodological development allows simultaneous revelation of the antigenic, landmark and proteomic maps on each immunoblot. A computer-assisted process using commercially available software automatically leads to the superimposition of the different maps, ensuring accurate localization of antigenic spots of interest.

## Introduction

Immunofluorescence, enzymatic and immunoprecipitation assays have been widely used for the identification of biomarkers in various diseases. These conventional methods are frequently based on immunoassays performed with limited antigenic targets, the choice of which has been driven by a supposedly well known pathophysiological rationale. Advances in proteomic methodologies (*in vitro* gene expression, 2D electrophoresis (2DE) and mass spectrometry) have allowed the emergence of standardized broad spectrum analysis methods such as serological analysis of recombinant tumor cDNA expression libraries (SEREX) and serological proteomic analysis (SERPA). These approaches have been developed to overcome the limitations of conventional methods. Based on a “without any *a priori*” strategy, they offer a simultaneous analysis of a wide range of reactivities, which surpasses the physiopathogenic hypotheses and offers an integrative interpretation of results.

SERPA, also called PROTEOMEX (an abbreviation of proteomics and SEREX) or SPEAR (serological and proteomic evaluation of antibody responses) [1], is a popular method that is judged to be reproducible and broadly applicable. It consists of combined proteomic approaches for the separation of proteins of interest and the serological screening of human serum antibodies. In contrast to SEREX, SERPA offers the advantage of considering the post-translational modifications in the natural context of protein expression. SERPA has been extensively used in different conditions and animal models such as allergic [2] and autoimmune [3–6] diseases but also in other conditions: cancerology [7–9], metabolism [10], toxicology [11] and infectiology [12–18]. However, after a decade of using SERPA, some methodological problems have emerged and must be emphasized. As observed for the proteomic approach [19], data obtained by SERPA have revealed the identification of redundant biomarkers in very different and unrelated disease conditions. A ‘hit-parade’ of the five most frequently reported targets of specific reactivities by SERPA in humans comprises: anti-enolase, anti-isomerase [4,6,8,20–32], anti-heat shock proteins [4,6,8,23–29,33–37], anti-heterogeneous nuclear ribonucleoprotein [8,28–31,38–41] and anti-peroxiredoxin [8,25–29,32,42] families. For example, specific anti-*α*-enolase reactivity has been described both in autoimmune diseases (multiple sclerosis [4], systemic sclerosis [29,37], autoimmune hepatitis [31,43,44], type 1 diabetes [32], rheumatoid arthritis [45,46], celiac disease [47] and Behçet’s disease [48,49]) and in different types of cancer (breast cancer [8,34], lung cancer [25,26], colorectal cancer [27], melanoma [41] and leukemia [50]). Only in rheumatoid arthritis has the specificity of anti *α*-enolase been confirmed, linked to the deimination of the target [46]. Such redundant identification could be explained, at least in part, by the interpretation biases linked to the superimposition of images of 2-DE immunoblots and gels. While these issues have been resolved in proteomics by differential in-gel electrophoresis (2D-DIGE) [51], these steps remain a major limitation in SERPA [52] even if different strategies have been proposed to circumvent them. These approaches are based either on the generation of a map of fixed benchmarks that offers additional visual anchors (so-called “landmark map” [4]) or the revelation of the proteomic map onto each immunoblot [34,36,53–55]. Even if they help final superimposition of the antigenic and the proteomic maps to target the protein spot to excise, these approaches still remain insufficient due to the persistence of an operator-dependent step to superimpose the different maps.

We propose here a new strategy, named ‘Fluorescence-based bidimensional immunoproteomic’ (FBIP) approach, derived from the 2D-DIGE procedure to ensure the accurate superimposition of the different maps. This methodology uses fluorescent probes to simultaneously reveal three maps on each immunoblot: the antigenic, the landmark and the proteomic maps. The superimposition stage now becomes an automated step based on a computer-assisted process using commercially available software.

## Materials and Methods

### Serological Antibodies

In order to illustrate our procedure, we analyzed the self IgG antibody responses with sera from 2 patients suffering from systemic lupus erythematosus. All patients gave their written informed consent and the study was approved by the local ethics committee (DC-2008-642; CHRU, Lille 2 University, France).

### Commercial Antibodies

Mouse monoclonal antibodies (mAbs) recognizing triosephosphate isomerase (TPIS, catalog reference TPI, H-11), glyceraldehyde phosphate dehydrogenase (GAPDH, catalog referenceA-3), *β*-actin (ACTB, catalog reference ACTBD11B7), *α*-enolase (ENOA, catalog reference A-5), heat shock 70 kDa protein (HSP71, catalog reference 3A3) (Santa Cruz Biotechnology, Inc., Santa Cruz, CA, USA) were used as a benchmark on the 2D immunoblots, providing anchors for the inter-assay alignment by generating a landmark map. Their localization on different proteomic maps has been extensively described (http://www.uniprot.org). The selection of these benchmarks was based on both their large pI and MW distributions on the proteomic map.

### Cell Cultures and Treatments

The human epithelial line cell HEp-2 from the American collection of cell cultures (ATCC CCL-23) was used for the experiments. HEp-2 cells were cultured in minimum essential medium (MEM) supplemented with 2mM L-glutamine, penicillin/streptomycin (50 units/mL), 1% sodium pyruvate, 1% non-essential amino acids and containing 10% (v/v) heat-inactivated fetal calf serum (FCS) (MEM-10% FCS) (Life technologies, Carlsbad, CA, USA). Cells were maintained at 37°C in a saturated humidity atmosphere containing 95% air and 5% CO2 in 75-cm^2^ flasks. For cell culture experiments, HEp-2 cells were detached by means of Trypsin/EDTA solution (Life technologies), re-suspended in MEM-10% FCS. After centrifugation at 1500 rpm for 10 min, numeration was performed using Scepter (Merck Millipore, Darmstadt, Germany), and cells were washed in PBS and then seeded at a concentration of 2x10^6^ cells per 75 cm² flask.

### Two-Dimensional Electrophoresis (2DE)

#### Protein extraction

The cell homogenization was performed as previously described [56]. Briefly, 5x10^6^ cells were homogenized in lysis buffer (8M urea/2M thiourea (GE Healthcare, Uppsala, Sweden), 4% CHAPS (Sigma Aldrich, Saint Louis, MO, USA), 50 mM DTT, anti-protease cocktail (Roche Diagnostics, Mannheim, Germany)) before protein precipitation using the 2D clean-up kit (GE Healthcare). The supernatant was removed and the pellet was suspended in 250 *μ*l of sample buffer (8M urea/2M thiourea, 4% CHAPS). The protein concentration was determined by Bradford assay (Bio-Rad Laboratories, Hercules, CA, USA).

#### Protein labeling

This step was exclusively used for the FBIP approach. Fifty micrograms per strip of HEp-2 extract protein were labeled with 400 pmol Cy3 dye (GE Healthcare), according the manufacturer’s instructions. Cy5 dye labeling was used when a post-staining step with Deep Purple (DP, GE Healthcare) was performed.

#### Isoelectrofocusing

Five hundred micrograms of proteins (of which 50 *μ*g was labeled for FBIP approach) were resuspended in 350 *μ*l of rehydration buffer composed by 8M urea/2M thiourea, 2% CHAPS, DeStreak Reagent (15 mg/ml, GE Healthcare) and ampholytes (1% IPG buffer, GE Healthcare). Sample loading for the first dimension was performed by passive in-gel rehydration. Proteins were first separated according to their isoelectric point (pI) along a non-linear (NL) immobilized pH gradient (IPG) strip (pH 3–11NL, 18 cm long; GE Healthcare) using the IPGphor III apparatus (GE Healthcare) for a total of 40,000 V.h.

#### SDS-PAGE electrophoresis

After focusing, the IPG strips were equilibrated for 15 min in a buffer 1 containing 6 M urea, 2% SDS, 30% glycerol, 50 mM Tris/HCl pH 8.6 and 1% DTT, and then for 15 min in buffer 2, in which the DTT in buffer 1 was replaced by 4.7% iodoacetamide. For the second dimension, equilibrated strips were loaded onto homemade 8–18% gradient polyacrylamide gels and overlaid with agarose solution (0.5% low-melting agarose and a trace amount of bromophenol blue). Electrophoresis was performed for 13–14 h in EttanDalt Six (GE Healthcare) with power limited to 3 W per gel. Among the 6 performed gels, one was used for Coomassie colloidal blue (CCB) staining.

### Blotting

Gels were blotted onto Hybond-ECL membranes (GE Healhtcare) using a “semi-dry” protocol (0.8 mA per cm²) and a discontinuous buffer system [57] for 2 hours.

### Immunoblotting

#### SERPA

Membranes were saturated with 5% non-fat dried milk. Western blotting was carried out with total sera (1/100). One membrane per patient’s serum has to be used in order to analyze the related antigenic map. For each study, one membrane is dedicated for the landmark map using a pool of commercial mAbs (anti-HSP71, 1/1,500; anti-ENOA, 1/5,000;anti-ACTB, 1/750; anti-G3P, 1/750; anti-TPIS, 1/200,000). Sera and commercial mAbs were diluted in Tris buffer saline (TBS: 100 mM Tris, pH 8.0; 0.3 M NaCl) containing 0.5% Tween20 (w/v) and 5% non-fat dried milk. After incubation overnight at 4°C, each antigenic map was revealed with an anti-human IgG horseradish peroxidase (HRP)-conjugated antibody 1/5,000 (Sigma Aldrich) and the landmark was revealed by HRP-conjugated anti-mouse IgG antibody 1/5,000 (Sigma Aldrich). All fluorograms were prepared using an enhanced chemiluminescence kit (GE Healthcare).

#### FBIP approach

After the step involving saturation of the membranes by 5% non-fat dried milk, the FBIP procedure allows one step co-hybridization of human serum defining the antigenic map and commercial antibodies defining the landmark map. After incubation overnight at 4°C, serum IgG reactivities were revealed with an anti-human IgG HRP-conjugated antibody 1/5,000 (Sigma Aldrich), and commercial mAbs were revealed using a donkey anti-mouse Alexa Fluor (AF) 647-conjugated Ab (Life Technologies). The peroxidase activity was revealed using an enhanced chemifluorescence kit (ECL PLUS, Pierce Biotechnology, Thermo Scientific, Sunnyvale, CA, USA). The 3 maps of each immunoblotted membrane were read simultaneously on a Typhoon 9400 scanner (GE Healthcare) using 3 coupled excitation and emission wavelengths: 532/580, 633/670, 488/520 for the proteomic (Cy3), landmark (AF647) and antigenic (ECL PLUS) maps, respectively. To take into account the potential MW shift related to the dye labeling, we performed a complementary coloration of the proteomic map by DP.

### Image Analysis Procedures

#### SERPA

The landmark and antigenic maps produced by the different 2D immunoblotting procedures were superimposed using visual parameters by zonal translation. The landmark map helped with superimposing the serum immune patterns on the CCB-stained proteomic map.

#### FBIP approach

The three 2D immunoblot maps simultaneously revealed by the Typhoon 9400 scanner were visualized immediately after capture with ImageQuant TL software (GE Healthcare). Multichannel revelation in the 3 fluorescent wavelengths allowed direct, operator-independent superimposition of both the antigenic and landmark maps on the proteomic map. This step constituted the “intra-assay alignment” step. Next, Progenesis SameSpots software (Totallab, Newcastle upon Tyne, England) was used for the process of inter-assay alignment between the different antigenic maps. Based on the 2D-DIGE alignment process, the proteomic maps used as internal standard were first aligned using correction vectors proposed by Progenesis SameSpots. After validation, the correction vectors defined for each proteomic map were automatically applied to each antigenic and landmark map associated with its own proteomic map. By comparing the landmark maps generated on each 2D immunoblot we were able to refine the strict superimposition of the different proteomic maps. This procedure, using a spot-by-spot approach, comparing both in-between serum reactivity and landmark map and serum profiles revealed whether or not they shared antibody reactivities.

### In-Gel Digestion and LTQ-ORBITRAP-Velos MS Analysis

MS protein identification procedures are detailed in Supporting Information (S1 Appendix).

Briefly, selected spots on the CCB-stained gel were manually excised (OneTouch 2D gel spot picker, Gel Company, San Francisco, CA, USA) and placed in a 96-well microtiter plate and conserved at -20°C. In-gel digestion and sample preparation were performed using the Freedom Evo 100 automation system (Tecan, Männedorf, Switzerland). Digest peptides were separated on a C18 nanochromatography column (RSLC Dionex, Thermo Scientific) directly coupled to a mass spectrometer (LTQ-OrbitrapVelos, Thermo Scientific). Using peak lists of peptide fragmentation fingerprint (PFF) and Proteome Discoverer 1.3 software (ThermoScientific) led to the identification of protein by querying the MASCOT (MatrixScience) search engine.

The global experimental design of FBIP approach is illustrated in Supporting Information (S1 Fig).

## Results

### Targeting of Antigenic Spots using Chemiluminescence Based Bi-Dimensional Immunoproteomic Approach: results and limits

**Fig 1:**
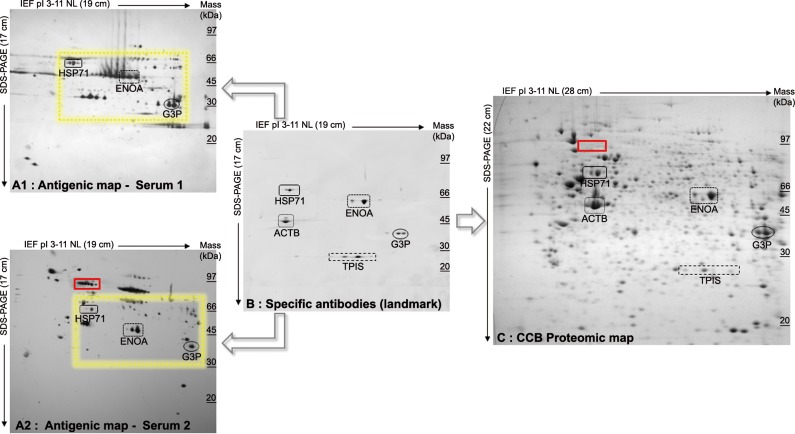
Chemiluminescence-based bidimensional immunoproteomic approach. After bi-dimensional electrophoresis of HEp-2 protein extract, we performed immunoblotting with sera from 2 patients with autoimmune diseases, thereby generating 2 antigenic maps (A, serum 1 and serum 2). We performed another immunoblotting (landmark map, B) using commercially available monoclonal antibodies against ACTB, HSP71, ENOA, G3P and TPIS. This landmark allowed anchoring to be used to superimpose both the antigenic maps (A) and the colloidal blue-stained proteomic map (C). The yellow area highlights some of the reactivity diversity between the 2 sera. The red area highlights antigenic spots (A, serum 2) for which there are no corresponding protein spots on the CCB gel (C). Sizes of immunoblots/gel and molecular weight distribution are indicated, to point out the distortions between their respective derived images. The differences between the relative mobility of HSP71 and G3P were measured at 3.6, 3.3 and 5.3 cm, respectively with serum 1, serum 2 and on CCB gel.

SERPA is based on the comparison of multiple images derived from distinct hybridizations obtained from different 2D immunoblotting procedures (Fig 1). In order to illustrate this procedure, we used selected sera from 2 patients suffering from an autoimmune disease. To facilitate the superimposition of these different antigenic maps (Fig 1A), we used a mix of commercially available monoclonal Abs directed against proteins (ACTB, HSP71, ENOA, G3P and TPIS) widely distributed in cellular extracts as for instance in HEp-2 cell line (Fig 1B). These commercial mAbs defined an antigenic landmark and were used as anchors to enable spot matching between the 2D immunoblots (Fig 1A) and the CCB proteomic map (Fig 1C).

Despite using this landmark map, we identified several limitations associated with this procedure. The first limitation was related to distortion of the final images (Fig 1A and 1C). The distortions are linked to the electrophoresis separation, as illustrated by differences in the distribution of the molecular weight standards and spots between the images. As shown in Fig 1A, the difference between the relative mobility of HSP71 and G3P spots was measured at 3.6 cm and at 3.3 cm on the antigenic maps respectively revealed with the serum 1 and 2. Moreover, the immunoblotting and staining procedures induced size modifications of the images, at a mean 19x17 cm and 26x22cm (length x height), respectively. On the proteomic map, the relative mobility of HSP71 and G3P spots was measured at 5.3 cm (Fig 1C). Secondly the serological hybridizations (Fig 1A) showed heterogeneous patterns containing from 10 to 300 antigenic spots, whereas CCB stained gel (Fig 1C) showed more than 1,000 protein spots distributed in the same ranges of pI and MW. Thirdly, we observed a strong heterogeneity of antigenic patterns between themselves (Fig 1A sera 1 and 2). In a particular region of the antigenic maps obtained (yellow area), we numbered 192 and 78 spots of reactivity with sera 1 and 2 respectively. Fourthly, the aspects of the antigenic spot could be visually different between them (HSP71, ENOA and G3P areas, Fig 1A, serum 1 and 2 & Fig 1B) and also with its own protein counterpart on the CCB gel (Fig 1C). Fifthly, due to the weak sensitivity of the CCB staining of the protein spots, each antigenic spot could not have its counterpart in the stained gel (red area, Fig 1A serum 2 and Fig 1C).

### Development of a New Method of SERPA using a Fluorescence-Based Bi-Dimensional Immunoproteomic approach (FBIP)

**Fig 2:**
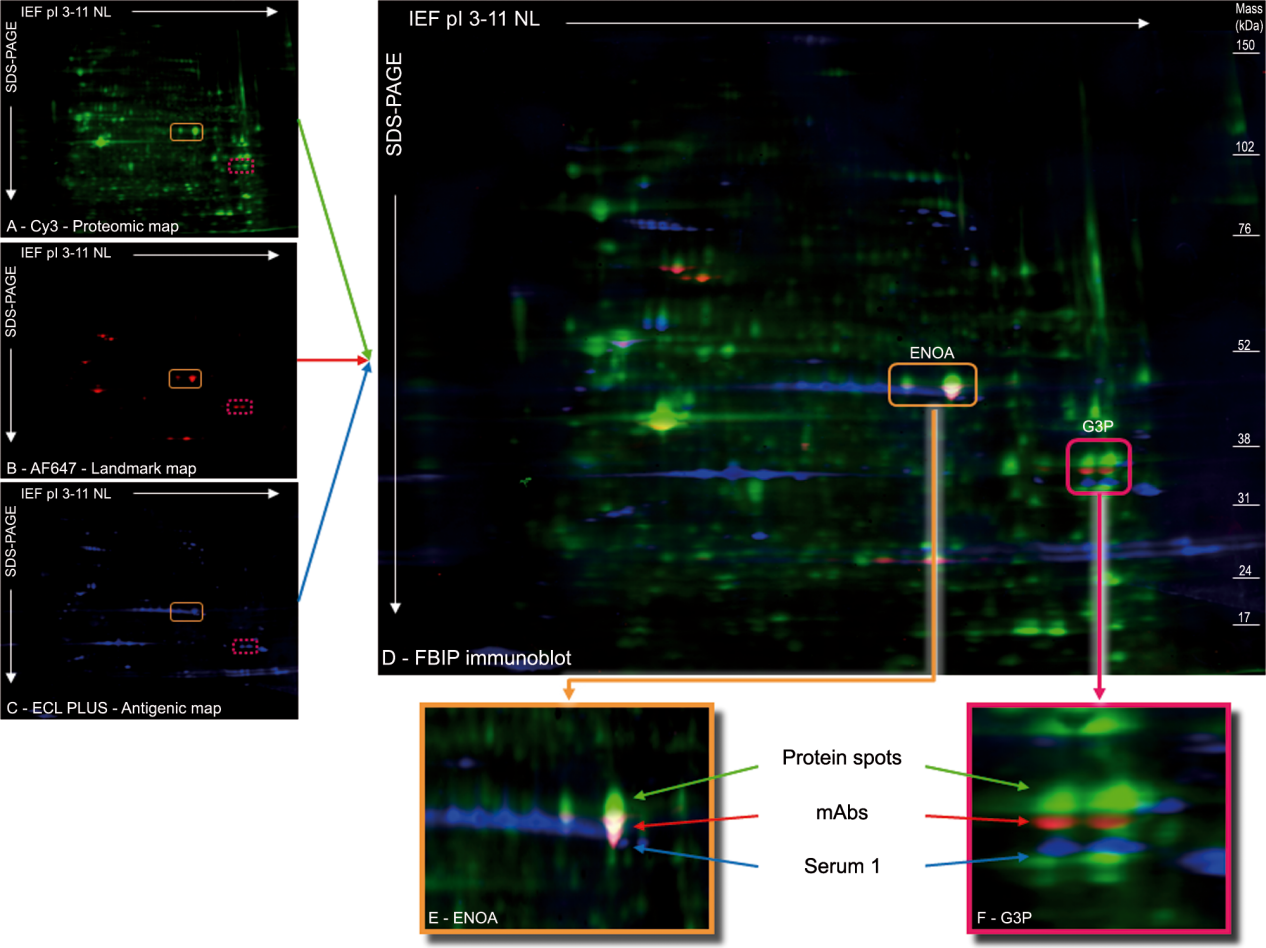
Fluorescence-based bidimensional immunoproteomic (FBIP) approach. In the FBIP procedure, 3 maps are generated from one 2D immunoblot (A–C). After 2-D electrophoresis of Cy3 labeled proteins (A), the immunoblotting is treated at the same time with a pool of commercially specific antibodies (landmark) and with human serum (antigenic map). The landmark is revealed with a set of Alexa Fluor 647-conjugated secondary antibodies (B). The antigenic map obtained with serum 1 is revealed with a horseradish peroxidase-conjugated anti-human antibody using chemifluorescence kit ECL PLUS (C). The direct superimposition of the 3 maps in the 3 wavelengths (D) revealed correct matching of reactivity towards ENOA (E) and a mismatch for reactivity against G3P (F). A defect of the superimposition was observed between the Cy3-labeled G3P protein and the corresponding mAb reactivity. The reproducibility of this approach is illustrated in the S2 Fig.

To normalize the superimposition procedure for the different antigenic maps, we used a cyanine-labeled proteomic map, which revealed the protein spots in the immunoblots. This permanently labeled proteomic map was repeated on every membrane in order to be revealed after 2D immunoblotting along with the antigenic and the landmark maps. It could then be used as an internal standard. To illustrate this procedure, we revealed the HEp-2 protein spots by Cy3 labeling (Fig 2A) and used the commercially available mAbs previously described and the same serological samples (illustrative data with serum 1).The commercial mAbs, used as anchors in the landmark map, were simultaneously revealed with a set of Alexa Fluor 647-conjugated secondary antibodies, associated with the Cy5 channel (Fig 2B). The antigenic map was revealed with an HRP-conjugated anti-human antibody using chemifluorescence kit ECL PLUS associated with the Cy2 channel (Fig 2C). This design allowed direct superimposition (Fig 2D) of the antigenic map on the proteomic map. To illustrate the advantages of this approach, we used a serological sample thought to contain both anti-ENOA and anti-G3P Abs as suggested by SERPA (Fig 1). The FBIP procedure revealed a correct co-alignment of the antigenic-, anchor- and protein spots for ENOA (Fig 2E). It revealed one mismatch between the antigenic and the anchors spots for G3P, suggesting a wrong association between the serological reactivity and the antigenic target by SERPA (Fig 2F).

Moreover we observed an absence of superimposition of the landmark- and the protein spots for G3P, suggesting a shift in molecular mass of Cy3 labeled proteins in the proteomic map (Fig 2F).

### Evidence of a Molecular Weight Shift Linked to the Cy3 Protein Labeling

**Fig 3:**
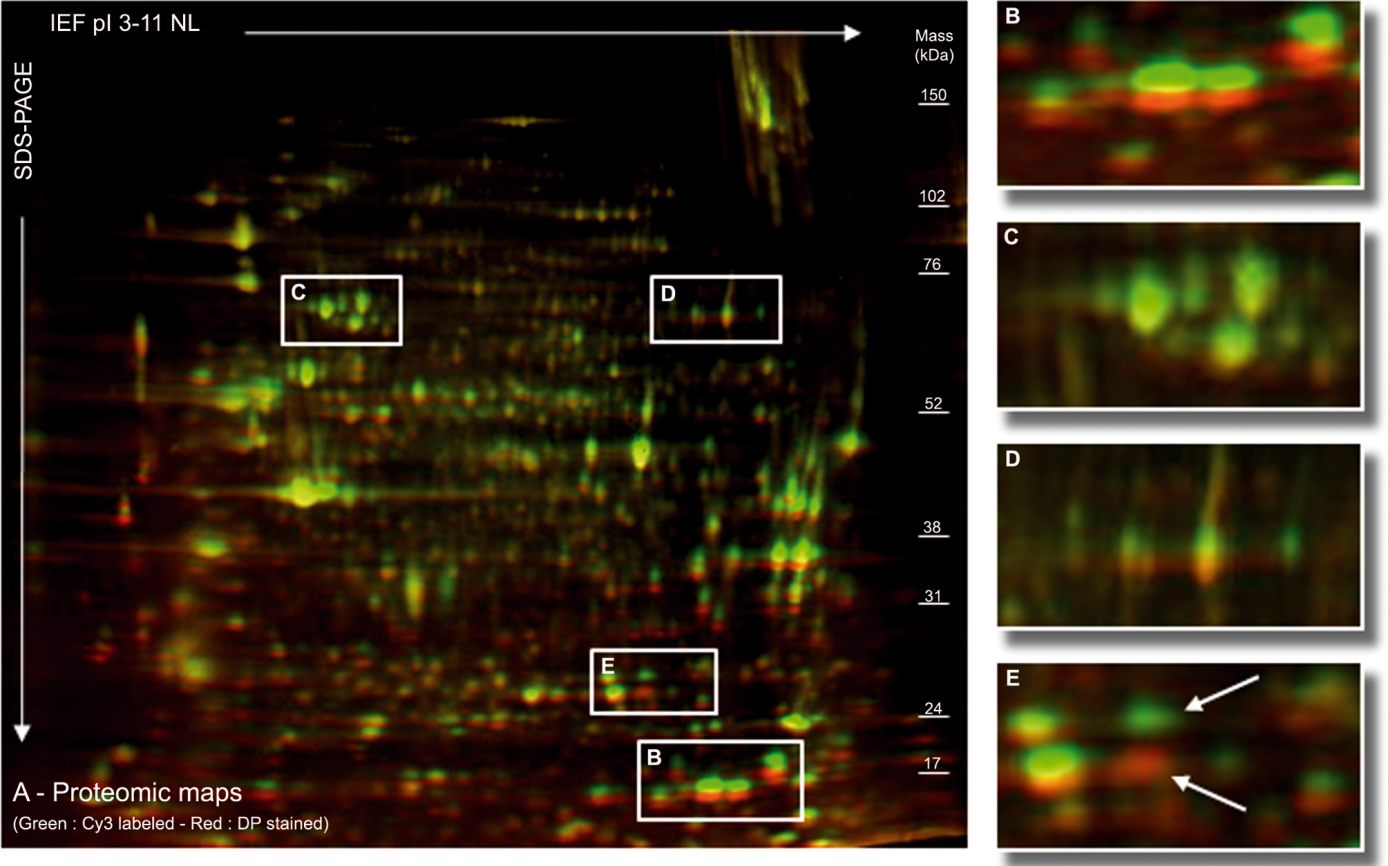
Assessment of heterogeneous molecular weight shift of proteins revealed by deep purple (DP) staining. The proteomic map has been labeled by Cy3 (green) and stained DP (red) (A). This process revealed a predominant shift of low molecular weight proteins, with the Cy3-labeled proteins appearing heavier than the DP-stained proteins (B). The shift seemed weaker and could even disappear in the case of higher molecular weight proteins (C) but this observation did not apply to all high MW proteins (D). Moreover, some proteins were stained only by Cy3 and others only by DP (arrows in E).

To illustrate the molecular weight shift between the 95% of non-labeled and the 5% of Cy3 labeled protein fractions, we performed a reversible DP staining of the protein map on the immunoblot (Fig 3A). This procedure revealed a heterogeneous shift, linked to the weak molecular weight of labeled protein (Fig 3B) or not (Fig 3C and 3D). Moreover, we observed that some proteins were labeled by Cy3 dye but not by DP, whereas other were labeled by DP but not Cy3 (Fig 3E).

### Correctives for Analysis of FBIP Immunoblots

**Fig 4:**
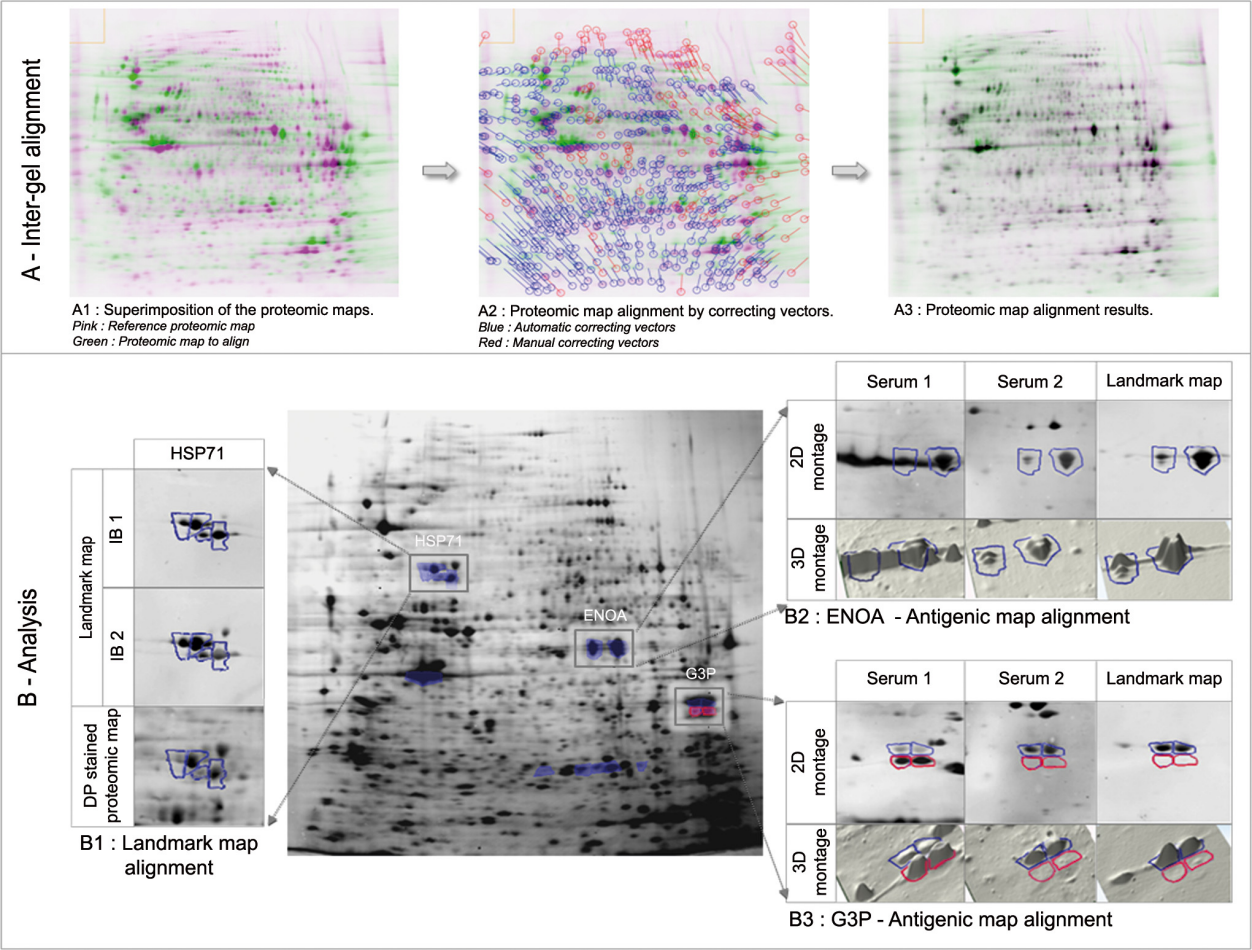
Progenesis SameSpots assisted analysis of FBIP approach. For the analysis of the immunoblots, Progenesis SameSpots software begins by inter-gel alignment step (A). To rectify the defect of superimposition of the proteomic maps revealed on each immunoblot (A1), Progenesis SameSpots suggested correcting vectors (blue vectors in A2). This automatic alignment was improved by applying manual vectors (red vectors in A2) to obtain perfect spot alignment (A3). For the analysis of immune reactivity, Progenesis SameSpots software displays both 2D and 3D images of reactivity associated with protein spots (B). The verification of the co-alignment of all the landmark maps between all the studied gels, and also on the DP stained proteomic map (i.e. HSP71 in B1) is the first step of analysis. Applied to the analysis of the reactivity of the 2 sera from patients with autoimmune diseases, this procedure demonstrated the co-alignment of antigenic, anchor and proteomic spots identified as ENOA by MS/MS (blue circles in B2), thus confirming that these patients had anti-ENOA autoantibodies. Using this procedure to analyze the anti-G3P (blue circles in B3) reactivity of the same 2 sera, we confirmed the data of SERPA for serum 2, which had anti-G3P autoantibodies. For serum 1, whereas SERPA had suggested a strong reactivity against G3P, FBIP approach revealed a weak reactivity against this target (blue circles in B3), but strong reactivity against smaller protein targets neighboring the G3P (red circles in B3), highlighting a clear mismatch of the SERPA procedure. Additional samples analyzed with the same procedures demonstrate the robustness of the FBIP approach (S3 Fig).

The analysis of FBIP immunoblots was supported by Progenesis SameSpots software initially designed for 2D-DIGE experiments. Using the Cy3-labeled proteomic map, considered as the internal standard, Progenesis SameSpots software suggested a panel of correcting vectors allowing the superimposition of all proteomic maps in order to take into account the potential migration defects (Fig 4A). Using these suggested correcting vectors defined in the Cy3 channel, the same parameters were automatically applied for the superimposition of the related landmark and antigenic maps, in the Cy5 and the Cy2 channels, respectively. In an ultimate step, the co-alignment of the anchors of the landmark maps on the expected protein spots was used as a final validation of the superimposition. The superimposition between the different maps was confirmed when the designated area delineating the protein spots strictly contained the landmark ones (Fig 4B1).

The FBIP immunoblot coupled with the Progenesis SameSpots analysis was used to analyze the reactivities of the same two sera previously studied by SERPA. This procedure demonstrated that co-reactivities were shared by the two sera. As previously described with SERPA (Fig 1), we observed a co-alignment with the anti-ENOA anchor (Fig 4B2) that was identified by MS/MS (Table 1). For serum 2, FBIP analysis also confirmed the G3P autoreactivity previously observed with SERPA. By contrast, as expected for serum 1, the previously observed mismatch (Fig 2F) was highlighted after Progenesis SameSpots analysis (Fig 4B3). Even though FBIP approach revealed a weak anti-G3P reactivity for serum 1, it was not consistent with the reactivity observed with SERPA, which matched with antigenic spots of lower molecular weights identified as hnRNPs by MS/MS (Table 1).

**Table 1:** List of proteins identified by LTQ-ORBITRAP-Velos.

Spot number^a^	Accession number^b^		Protein name^b^	Gene name^b^	pI^c^ Theo	pI^c^ Exp	MW (kDa)^c^ Theo	MW (kDa)^c^ Exp	Number of identified peptides	Total Ion Score	Sequence coverage (%)
1	P06733	ENOA_HUMAN	Alpha-enolase	ENO1	7	6.8	47	47	23	1392	69
2	P06733	ENOA_HUMAN	Alpha-enolase	ENO1	7	7	47	47	24	8133	71
3	P04406	G3P_HUMAN	Glyceraldehyde-3-phosphate dehydrogenase	GAPDH	8.6	8.5	36	35	13	4152	54
4	P04406	G3P_HUMAN	Glyceraldehyde-3-phosphate dehydrogenase	GAPDH	8.6	8.7	36	35	15	5692	55
5	P22626	ROA2_HUMAN	Heterogeneous nuclear ribonucleoproteins A2/B1	HNRNPA2B1	9	8.5	37	33	13	3157	44
6	P22626	ROA2_HUMAN	Heterogeneous nuclear ribonucleoproteins A2/B1	HNRNPA2B1	9	8.7	37	33	13	4122	44

^a^Spot number according in Fig 4B2 and 4B3.

^b^Accession number and protein name according SwissProt.

^c^Isoelectric point (pI) and molecular weight (MW): Theoretical (Theo) and Experimental (Exp).

## Discussion

We have described here a co-detection system based on a fluorescent bi-dimensional immunoproteomic approach which allowed the exact location of protein that supported the specific reactivities of interest. This procedure is derived from 2D-DIGE using CyDye-labeled protein maps, which is repeated on each 2D immunoblot as internal standard. This procedure, which includes both landmark and antigenic maps, respectively revealed by an Alexa Fluor coupled secondary Ab and chemifluorescence, allows simultaneous visualization of the three maps: proteomic, landmark and antigenic. It offers the guarantee of strictly superimposing antigenic spots on the protein map if the potential molecular shift induced by the CyDye labeling is considered with complementary staining using DP. This FBIP approach is thought to improve the classical serological proteomic approach.

SERPA has been extensively used in different scientific and medical fields, and especially for identifying new biomarkers in cancerology [7,8], allergology [2], autoimmunity [3–6,11] and vaccination strategy [12,15,16]. SERPA has led to the identification of targets described as biomarkers in diseases. However, some specific biomarkers could be shared by different and unrelated pathological processes, as observed with proteomics [19]. These data raise questions about their relevance and may well explain the reduction in the number of reports published in recent years.

Based on our experience, we hypothesized that the identification of such common reactivities could be attributed, at least in part, to a critical step of the methodological approach: the superimposition of the antigenic and the proteomic maps. The conventional bi-dimensional immunoproteomic approach SERPA is based on comparative analyses of different proteomic and antigenic maps generated by separately performed distinct 2DE gels. As each of these gels undergoes different distortion constraints, the proteomic maps are stochastically distorted. The challenge of operator-dependent visual superimposition of the antigenic and proteomic maps is associated with the comparison of very different kinds of images whereas few as 50 antigenic spots may have to be matched on a proteomic map containing at least 1,500 protein spots. Moreover, the distortion constraints associated with the antigenic map are largely underestimated in relation to the few antigenic spots revealed. Together, these two methodological aspects, intrinsic to SERPA, could lead to mismatches and finally to wrong protein identification.

Although these methodological aspects have not been clearly described yet, different strategies have been employed to try to circumvent these problems. From 2004, we used commercially available antibodies directed against housekeeping proteins to generate a landmark map, containing anchors which facilitate the alignment of antigenic and protein spots [4]. This process was adopted by Farinazzo et al. and Goeb et al. [58,59]. However, the restricted number of spots generated and the manual stage of matching limited the correction efficiency of this approach. Several authors have reported post-staining of the membrane, using colloidal gold particles [34,37,53], silver [54,60] or CCB [55,61] after immunodetection. This approach presented the advantage of staining the proteomic map from each immunoblot, but it implied the treatment of different images generated from 2 distinct steps. For example, in the procedure applied by Canelle et al. [34], the antigenic map image was superimposed on its related colloidal gold-stained transferred protein map image. Then, the stained transferred protein map image was superimposed on the CCB-stained protein map image using Image Master 2D Platinium 5 software. The alignment of spots from the transferred protein map on the CCB-stained protein map allowed corrections of distortions between membrane and gels, making it easier to match the antigenic and protein spots. However, this supplementary step did not eliminate manual superimposition of the antigenic and transferred proteomic maps. Moreover, the sensitivity of these stains was intrinsically lower than that of the antibody detection system, leading to some matching defects between the antigenic and the transferred proteomic maps.

Some authors replaced post-immunostaining with fluorescent staining just after the transfer step [62], while others added the revelation of the immunoblots by chemifluorescence [63,64]. These approaches took advantage of using the same revelation system for the post-transferred protein and antigenic maps. But the superimposition of these 2 maps remained a manual procedure even if they used image analysis software (Adobe Photoshop) [63,64]. Some authors have also suggested adding a ‘pen’ landmark to facilitate the superimposition of the antigenic map on its related stained transferred protein map image [61]. The defect common to all these approaches was the generation of multiple images that had to be aligned manually. In the immunoblot 2D procedure, the use of fluorescent probes has previously been reported in different medical fields. Some authors used chemifluorescence to reveal the antigenic map [55,63,64]. By contrast, others have used fluorescent probes for protein labeling before the electrophoresis steps [65–67]. Goeb et al. [59] use a fluorescent dye for staining a landmark map in order to confirm the localization of antigenic spots as potential autoimmune targets. In all these works, the superimposition step remains visual.

We describe here a one-step procedure that generates 3 images intrinsically matched by scanning the same immunoblots with 3 different wavelengths. The manual alignment between the antigenic and the proteomic maps is bypassed by this procedure. Moreover, proteomic analysis software, using correcting vectors, automatically performed the alignment of all the labeled protein maps, thereby ensuring inter-gel matching. The alignment parameters of the protein maps are directly reported onto their related antigenic maps, thus ensuring the matching of the different immunoblots.

This procedure is derived from the 2D-DIGE technology, which automatically aligns different proteomic maps revealed at the same time using fluorescent probes read at different wavelengths. This approach uses an internal standard in order to match and normalize the protein profiles. A similar approach was used in a recently published study [68]. In our study we took advantage of the internal standard and transposed it as a repeated proteomic map on each immunoblot in order to guarantee the matching of the antigenic maps. In our FBIP procedure, we performed Cy3 labeling for the proteomic map associated to each immunoblot. However, we used fluorescent revelation systems with wavelengths similar to CyDye 2 and CyDye 5 for the antigenic and landmark maps, respectively. Moreover, using a landmark map based on an Ab detection system, our procedure reached the sensitivity scale of the antigenic map, thus providing the means to control the quality of the alignment parameters.

Some studies have applied multiplex fluorescent probes in 2D immunoblots in order to superimpose antigenic and proteomic maps [41,66,69]. In these studies, the proteomic map was labeled by CyDye when the antigenic map was revealed by other fluorescent probes. Suzuki et al. [41] and Katsumata et al. [69] performed post-blotting staining using CyDye monoreactive Dye pack. This staining presents the advantage of labeling membrane-fixed proteins, but in our experience this monoreactive dye is less sensitive than free CyDye and frequently induces a high background that could disturb the matching of antigenic and proteomic maps. In their procedure, Fontaine et al. [66] used 150*μ*g of labeled proteins as antigenic sample to detect serological reactivities during sample preparation. In our experience, to reveal such reactivities on cellular or tissue extracts, 500*μ*g of protein have to be loaded on gel as suggested by manufacturers (GE Healthcare) to perform “preparative 2D-DIGE gels”. Such a quantity optimized the revelation of immune reactivities whatever the affinity of Ab. In the experimental conditions, of “preparative 2D-DIGE gels”, manufacturers advise the optimal labeling of a fraction of protein sample to avoid too high a brightness and to enhance the resolutive separation of signals. In this context, we observed the well-known mass shift between the free labeled proteins and the CyDye stained ones. Whereas CyDye labeling was initially described as mainly affecting the low molecular weight proteins, we observed a more heterogeneous distortion of apparent molecular weight, likely related to the lysine distribution in the amino acid sequence of proteins. This mass shift was recently observed in another study using a similar labeling design [68]. However, the restricted focusing on a single antigenic target could not be expected to resolve this question. In a large immunoscreening approach and in order to circumvent this problem, we suggested introducing a reversible post-labeling of blotted proteins with DP staining.

This step was crucial to optimize the matching of antigenic and protein spots to be selected and took advantage of the use of inter-gel alignment generated by specific software, such Progenesis SameSpots (Totallab), Delta2D (Decodon GmbH, Greifswald, Germany) or Decyder (GE Healthcare). Such electronic tools aligned all CyDye stained proteomic maps, considered as internal standard, on both the different immunoblots and the DP stained membrane. In a second step, the software reported all the alignment correcting vectors on the antigenic maps, allowing their superimposition on the DP map. The alignment procedure of FBIP approach was totally operator free and could reveal mismatches of the SERPA alignment procedure partially based on the morphological aspects of antigenic and protein spots. This is illustrated in our data by the mismatch on anti-G3P reactivity and hnRNP spot protein.

If the analysis of 2D immunoblots is largely assisted by 2D-DIGE derived software, they showed limits linked to intrinsic properties of FBIP approach, mainly the qualitative exploitation of experimental data. In the 2D-DIGE procedure, the interpretation of data is based on the comparative expression level of every protein spot, normalized according to the internal standard. This generates a classification of data based on an expression differential, where 2D immunoblots should identify intragroup similar antigenic patterns, which will be submitted to intergroup variability analysis. For example, an antigenic spot recognized by several patients could be missed by such 2D-DIGE analysis software, while these data could illustrate a significant association of antigenic recognition specifically related to a disease condition. For FBIP approach, the qualitative analysis of the signal would be sufficient for the definition of each antigenic map, and the inter-assay comparison. Yet, the quantitative analysis of 2D-DIGE analysis software did not enable qualitative analysis in terms of presence or absence of antigenic spots on 2D immunoblots.

Moreover, such software performed statistical analysis using principal component analysis, where some other multiparametric statistical tests have to be used. Such approaches could justify exporting qualitative rough data in a spreadsheet format. Today, the software does not currently offer this function. Finally, a hardware solution is able to reveal up to 5 different channels of wavelengths, where the software only uses data generated by 3 wavelengths. For example, a five-channel procedure allows the analysis of the impact of post-translational modifications on protein antigenicity through a one-step method.

In summary, we have described an accurate method to circumvent interpretation biases related to the superimposition procedure of SERPA. Applied with fractionation procedures and the handling of IEF gels with restricted pI ranges which enhance the detection of low abundance antigens and also improve the resolution of 2D gels, this methodological evolution of FBIP analysis will improve the identification of pertinent biomarkers of diseases.

## Supporting Information

S1 Appendix Protein Identification by Mass Spectrometry (MS) and Database Searching. (DOCX)

S1 Fig Experimental design for an optimized Fluorecence-Based Immunoproteomic (FBIP) approach for accurate screening of autoantibodies. Step 1: The proteins (Hep-2 cells here) were extracted in lysis buffer (8M urea, 2M thiourea, 4% CHAPS, 50 mM DTT, anti-protease cocktail) and they were precipitated using the 2D clean-up kit. The protein concentration was evaluated by Bradford assay. For each gel, 50 μg were labeled with 400 pmol Cydye (Cy3 or Cy5). Before separation on two-dimensional (2-D) gels, 50 μg of labeled proteins were pooled with 450 μg of labeled-free proteins. Step 2: Proteins were electrofocused according to their isoelectric point (pI) along a non-linear immobilized pH gradient strip (pH 3–11NL, 18 cm long) using the IPGphor III apparatus(GE Healthcare) for a total of 40,000 V.h. After focalization, equilibrated strips were loaded onto homemade 8–18% gradient polyacrylamide gels and electrophoresis was carried out in an Ettan Dalt six (GE Healthcare). Gels were run overnight at 12°C at a constant power of 3 watts/gel until the bromophenol blue dye front reached the bottom of the gel. After 2-D electrophoresis, proteomic map labeled by CyDye can be visualized by Typhoon 9400 scanner (GE Healthcare). Step 3: In order to compare the Ab reactivities of (n) patients suffering from an auto-immune disease, (n) 2D gels were transferred onto nitrocellulose membrane. Membranes were immunoblotted with a set of commercial monoclonal antibodies (mAbs) (anti-HSP71, 1/1,500; anti-ENOA, 1/5,000; anti-ACTB, 1/750; anti-G3P, 1/750; anti-TPIS, 1/200,000) and simultaneously with a serum at 1:100 dilution. The commercial mAbs (Landmark) were revealed using a donkey anti-mouse Alexa Fluor (AF) 647-conjugated antibody. The patients Ab reactivities (here IgG) were revealed with a horseradish peroxidase (HRP)-conjugated antibody and an enhanced chemifluorescence kit (ECL plus). The 3 maps (proteomic, landmark and IgG reactivities maps) of each immunoblotted membrane were revealed in the same time on a Typhoon 9400 scanner using 3 coupled excitation and emission wavelengths: 532/580, 633/670, 488/520 respectively at 200 μm resolution. Step 4: To take into account the potential MW shift related to the dye labeling, one gel was transferred onto nitrocellulose membrane. This membrane was stained with Deep Purple (DP) and the two proteomic maps (one labeling by Cydye and the total proteomic map revealed by DP staining) were revealed at the same time on a Typhoon 9400 scanner using two coupled excitation and emission wavelengths: 633/670; 532/580 respectively at 200 μm resolution. Step 5: The image analysis software (Progenesis SameSpots) allows to align all labeled proteomic maps (used as an internal standard) by automatic and manual correction vectors. Finally the comparison of Ab reactivities generates a “spot picking list” which defines protein spots of interest targeted by patients Ab. Step 6: For protein identification to selected spots, after 2-D electrophoresis, one gel was stained with Coomassie colloidal blue (CCB). Step 7: Protein spots of interest were excised from CCB-gel and conserved at -20°C until digestion and identification by mass spectrometry.
(TIF)

S2 Fig FBIP reproducibility. Method reproducibility has been evaluated performing different FBIP procedures (independent IEF to SDS page steps) on several protein extracts issued from more than 3 different HEp-2 cell cultures. Here are illustrated 3 replicates (REP-1 to REP-3) of the FBIP procedure using the same serum. The reproducibility is estimated over to 80%. Arrows illustrated the punctual difference classically observed on antigenic maps but also on landmark maps.
(TIF)

S3 Fig Robustness of the FBIP alignment method. The capacity of analysis of the FBIP alignment method has been assessed through the analysis of different antigenic maps generated with large cohorts of patients. The association of automatic and manual correction vectors in combination with the verification step of the co-alignment of all the landmark maps focus the analysis of antigenic map on specific areas, independently of the presence of reactivity. Here are illustrated the referent DP stained proteomic map (A) and the anti ENO-A reactivity of 8 healthy subjects generated after Progenesis SameSpots analysis (B).
(TIF)
