# Supplementary material for: An Optimized Fluorescence-Based Bidimensional Immunoproteomic Approach for Accurate Screening of Autoantibodies
Source: PLoS One. 2015 Jul 1;10(7):e0132142. doi: 10.1371/journal.pone.0132142 (PMC4489013; doi:10.1371/journal.pone.0132142)
Supplement: S1 Appendix — (DOCX) [file pone.0132142.s001.docx]

**Supporting Information 1 : Protein Identification by Mass Spectrometry (MS) and Database Searching**

Spots of interest were manually excised. Spots excised from gels were placed into 96-well microtiter plates. Then, in-gel digestion was carried out with trypsin as described by Shevchenko et al. [1] with minor modifications and using for all steps a Freedom EVO 100 digester/spotter robot (Tecan, Switzerland). Spots were first destained two times with a mixture of 100 mM ammonium bicarbonate (ABC) and 50% (v/v) acetonitrile (ACN) for 45 min at 22°C and then dried using 100% ACN for 15 min. Protein spots were then reduced with 25 mM ABC containing 10 mM DTT for 1 h at 60°C and then alkylated with 55 mM iodoacetamide in 25 mM ABC for 30 min in the dark at 22°C. Gels pieces were washed twice with 25 mM ABC and finally shrunk two times with 100% ACN for 15 min and dried using 100% ACN for 10 min. Bands were finally completely dehydrated after 1 h at 60°C. Gel pieces were incubated with 13 μL of sequencing grade modified trypsin (Promega, USA; 12.5 μg/mL in 40 mM ABC with 10% ACN, pH 8.0) overnight at 40°C. After digestion, peptides were washed with 30 μL of 25 mM ABC, shrunk with 100% ACN and extracted twice with a mixture of 50% ACN–5% formic acid (FA). Extracts were dried using a vacuum centrifuge Concentrator plus (Eppendorf).

For MS and MS/MS LTQ-ORBITRAP analysis, analyses were realized using an Ultimate 3000 Rapid Separation Liquid Chromatographic (RSLC) system (Thermo Fisher Scientific) online with a hybrid LTQ-Orbitrap-Velos mass spectrometer (Thermo Fisher Scientific). Briefly, peptides were loaded and washed on a C_18_ reverse phase precolumn (3 µm particle size, 100 Å pore size, 150 µm i.d., 1 cm length). The loading buffer contains 98% H_2_O, 2% ACN and 0.1% trifluoroacetic acid (TFA). Peptides were then separated on a C_18_ reverse phase resin (2 µm particle size, 100 Å pore size, 75 µm i.d., 15 cm length) with a 4 min “effective gradient” from 100% A (0.1% FA and 100% H_2_O) to 50% B (80% ACN, 0.085% FA and 20% H_2_O).

The Linear Trap Quadrupole Orbitrap mass spectrometer acquired data throughout the elution process and operated in a data dependent scheme with full MS scans acquired with the Orbitrap, followed by up to 20 LTQ MS/MS CID spectra on the most abundant ions detected in the MS scan. Mass spectrometer settings were: full MS (AGC: 1*10^6^, resolution: 6*10^4^, m/z range 400-2000, maximum ion injection time: 500 ms); MS/MS (AGC: 5*10^3^, maximum injection time: 50 ms, minimum signal threshold: 500, isolation width: 2Da, dynamic exclusion time setting: 15 s). The fragmentation was permitted of precursor with a charge state of 2, 3, 4 and up. For the spectral processing, the software used to generate .mgf files is Proteome discoverer 1.3. The threshold of Signal to Noise for extraction values is 3.

Database searches were carried out using Mascot version 2.4 (MatrixScience, London, UK) on “Homo sapiens” proteins from SwissProt databank containing 20322 sequences (March 2013) (http://www.ncbi.nlm.nih.gov/). The search parameters were as follows: carbamidomethylation as a variable modification for cysteins and oxidation as a variable modification for methionines. Up to 1 missed tryptic cleavage was tolerated and mass accuracy tolerance of 5 ppm for precursors and 0.45 Da for fragments were used for all tryptic mass searches. Positive identification was based on a Mascot score above the significance level (i.e. < 5%).The reported proteins were always those with the highest number of peptide matches. Under our identification criteria, no result was found to match to multiple members of a protein family.

1. Shevchenko A, Loboda A, Ens W, Schraven B, Standing KG et al. (2001) Archived polyacrylamide gels as a resource for proteome characterization by mass spectrometry. Electrophoresis 22/6: 1194-1203.
