# Supplementary figures and images for: An Optimized Fluorescence-Based Bidimensional Immunoproteomic Approach for Accurate Screening of Autoantibodies
Source: PLoS One. 2015 Jul 1;10(7):e0132142. doi: 10.1371/journal.pone.0132142 (PMC4489013; doi:10.1371/journal.pone.0132142)

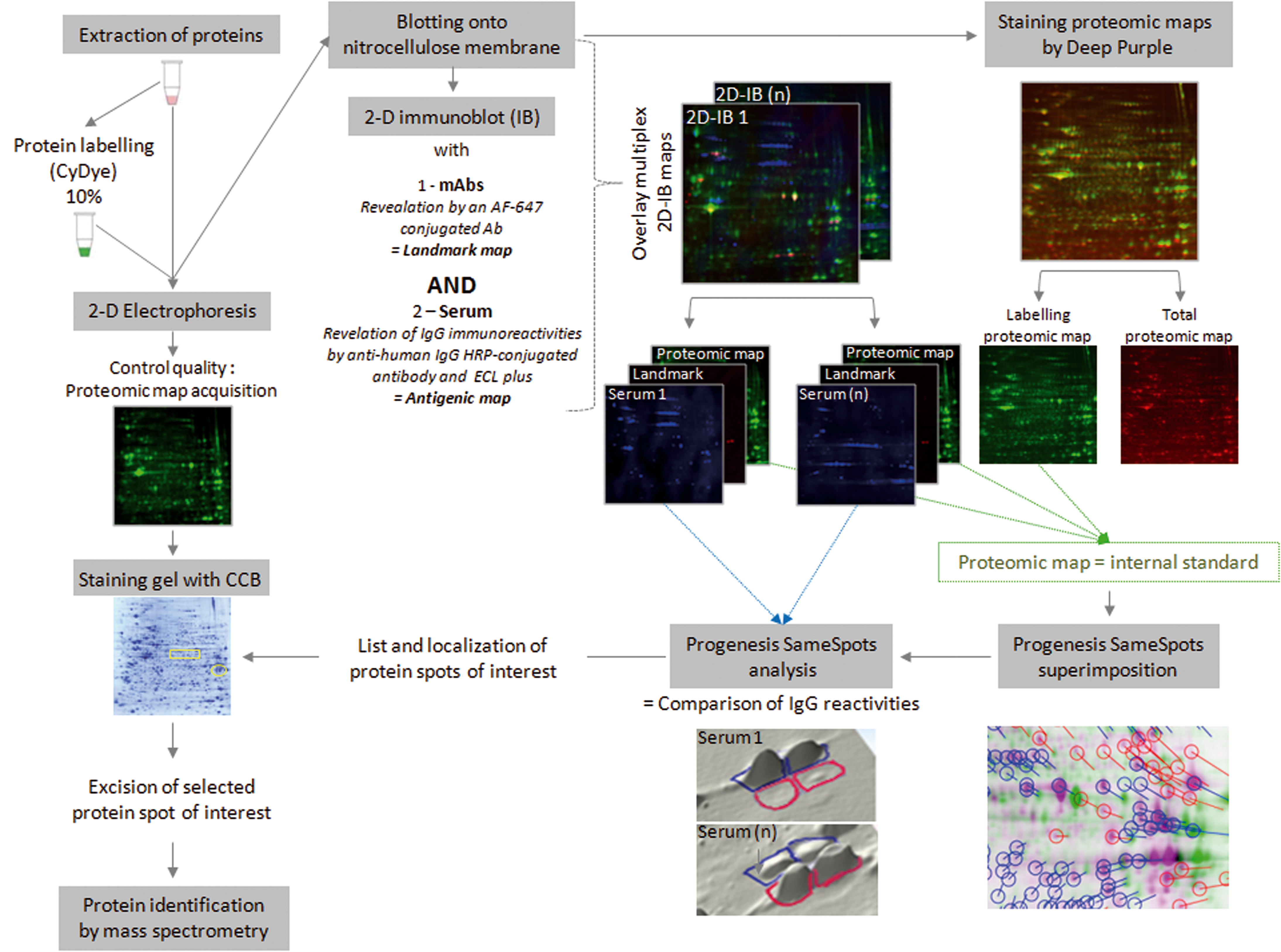

Supplement: S1 Fig — Step 1: The proteins (Hep-2 cells here) were extracted in lysis buffer (8M urea, 2M thiourea, 4% CHAPS, 50 mM DTT, anti-protease cocktail) and they were precipitated using the 2D clean-up kit. The protein concentration was evaluated by Bradford assay. For each gel, 50 μg were labeled with 400 pmol Cydye (Cy3 or Cy5). Before separation on two-dimensional (2-D) gels, 50 μg of labeled proteins were pooled with 450 μg of labeled-free proteins. Step 2: Proteins were electrofocused according to their isoelectric point (pI) along a non-linear immobilized pH gradient strip (pH 3–11NL, 18 cm long) using the IPGphor III apparatus(GE Healthcare) for a total of 40,000 V.h. After focalization, equilibrated strips were loaded onto homemade 8–18% gradient polyacrylamide gels and electrophoresis was carried out in an Ettan Dalt six (GE Healthcare). Gels were run overnight at 12°C at a constant power of 3 watts/gel until the bromophenol blue dye front reached the bottom of the gel. After 2-D electrophoresis, proteomic map labeled by CyDye can be visualized by Typhoon 9400 scanner (GE Healthcare). Step 3: In order to compare the Ab reactivities of (n) patients suffering from an auto-immune disease, (n) 2D gels were transferred onto nitrocellulose membrane. Membranes were immunoblotted with a set of commercial monoclonal antibodies (mAbs) (anti-HSP71, 1/1,500; anti-ENOA, 1/5,000; anti-ACTB, 1/750; anti-G3P, 1/750; anti-TPIS, 1/200,000) and simultaneously with a serum at 1:100 dilution. The commercial mAbs (Landmark) were revealed using a donkey anti-mouse Alexa Fluor (AF) 647-conjugated antibody. The patients Ab reactivities (here IgG) were revealed with a horseradish peroxidase (HRP)-conjugated antibody and an enhanced chemifluorescence kit (ECL plus). The 3 maps (proteomic, landmark and IgG reactivities maps) of each immunoblotted membrane were revealed in the same time on a Typhoon 9400 scanner using 3 coupled excitation and emission wavelengths: 532/580, 633/670, 488/520 respectiv [file pone.0132142.s002.tif]

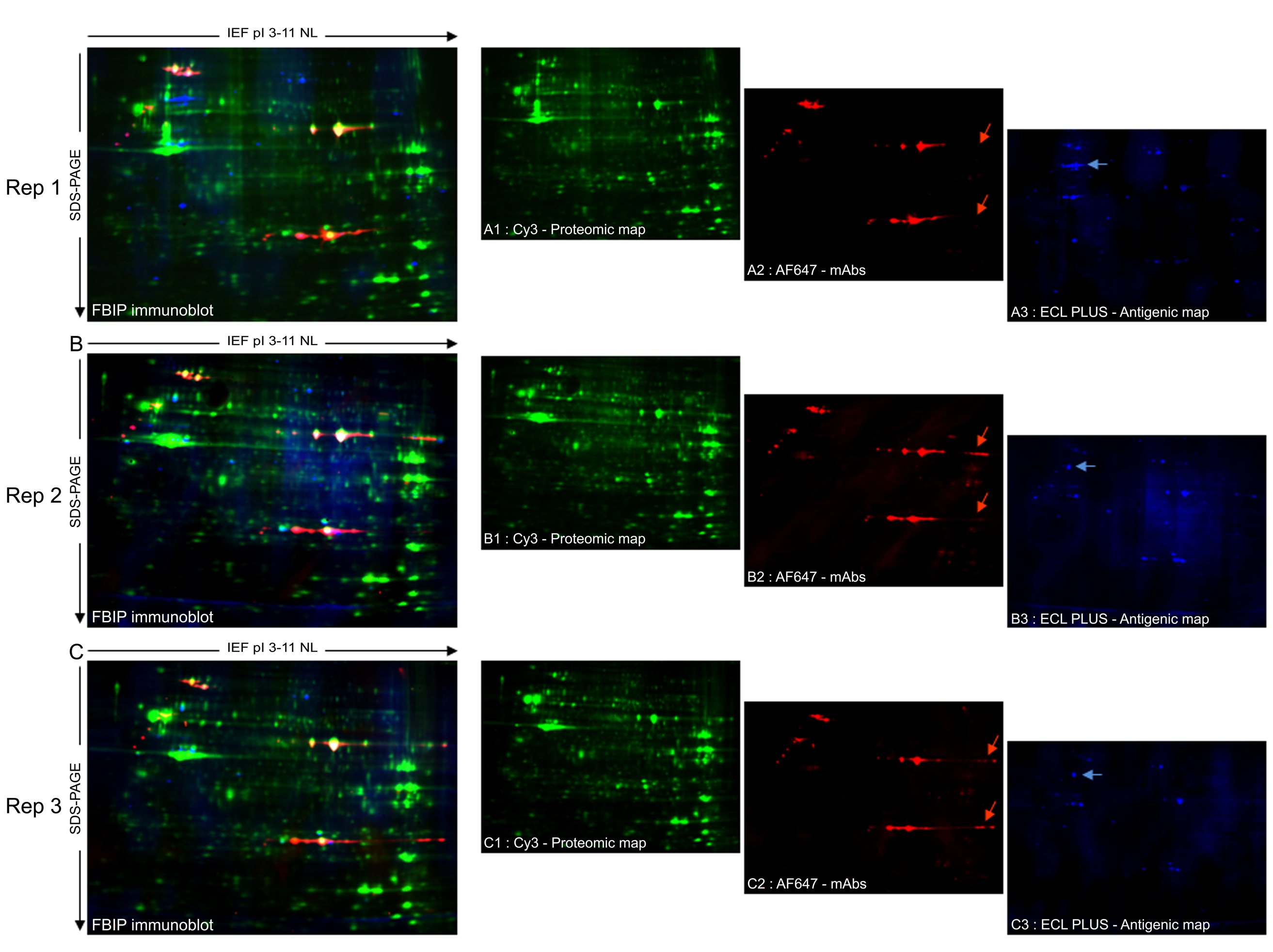

Supplement: S2 Fig — Method reproducibility has been evaluated performing different FBIP procedures (independent IEF to SDS page steps) on several protein extracts issued from more than 3 different HEp-2 cell cultures. Here are illustrated 3 replicates (REP-1 to REP-3) of the FBIP procedure using the same serum. The reproducibility is estimated over to 80%. Arrows illustrated the punctual difference classically observed on antigenic maps but also on landmark maps. (TIF) [file pone.0132142.s003.tif]

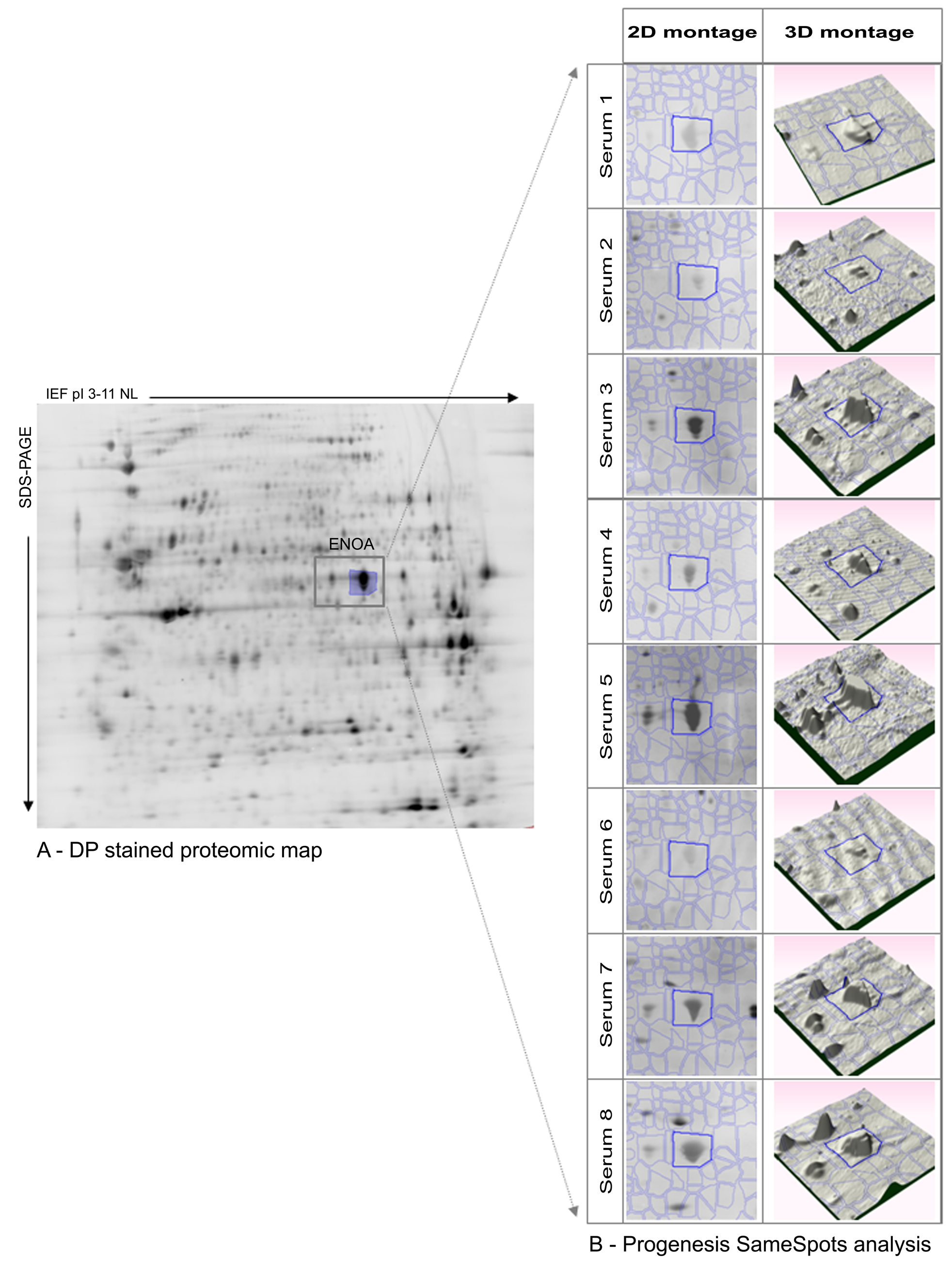

Supplement: S3 Fig — The capacity of analysis of the FBIP alignment method has been assessed through the analysis of different antigenic maps generated with large cohorts of patients. The association of automatic and manual correction vectors in combination with the verification step of the co-alignment of all the landmark maps focus the analysis of antigenic map on specific areas, independently of the presence of reactivity. Here are illustrated the referent DP stained proteomic map (A) and the anti ENO-A reactivity of 8 healthy subjects generated after Progenesis SameSpots analysis (B). (TIF) [file pone.0132142.s004.tif]
